# Virtual Reality Applications in Chronic Pain Management: Systematic Review and Meta-analysis

**DOI:** 10.2196/34402

**Published:** 2022-05-10

**Authors:** Lisa Goudman, Julie Jansen, Maxime Billot, Nieke Vets, Ann De Smedt, Manuel Roulaud, Philippe Rigoard, Maarten Moens

**Affiliations:** 1 Department of Neurosurgery Universitair Ziekenhuis Brussel Jette Belgium; 2 STIMULUS Vrije Universiteit Brussel Jette Belgium; 3 Center for Neurosciences Vrije Universiteit Brussel Jette Belgium; 4 Pain in Motion Research Group Department of Physiotherapy, Human Physiology and Anatomy Faculty of Physical Education and Physiotherapy Jette Belgium; 5 Research Foundation—Flanders Brussel Belgium; 6 PRISMATICS Poitiers University Hospital Poitiers France; 7 Department of Physical Medicine and Rehabilitation Universitair Ziekenhuis Brussel Jette Belgium; 8 Department of Spine Surgery & Neuromodulation Poitiers University Hospital Poitiers France; 9 Pprime Institute University of Poitiers Chasseneuil-du-Poitou France; 10 Department of Radiology Universitair Ziekenhuis Brussel Jette Belgium

**Keywords:** virtual reality, chronic pain, systematic review, multilevel meta-analysis, immersive technologies, clinical outcomes, mobile phone

## Abstract

**Background:**

Virtual reality (VR) is a computer technology that immerses a user in a completely different reality. The application of VR in acute pain settings is well established. However, in chronic pain, the applications and outcome parameters influenced by VR are less clear.

**Objective:**

This review aimed to systematically identify all outcome parameters that are reported in relation to VR in patients with chronic pain.

**Methods:**

A total of 4 electronic databases (PubMed, Scopus, Web of Science, and Embase) were searched for relevant studies. Multilevel random-effect meta-analyses were performed, whereby the standardized mean difference was chosen as the effect size to denote the difference between measurements before and after a VR intervention.

**Results:**

The initial database search identified 1430 studies, of which 41 (2.87%) were eventually included in the systematic review. Evidence has been found for the effects of VR on pain, functioning, mobility, functional capacity, psychological outcomes, quality of life, neuropsychological outcomes, and physical sensations. The overall effect size (a total of 194 effect sizes from 25 studies) based on a three level meta-analysis was estimated at 1.22 (95% CI 0.55-1.89; *z*=3.56; *P*<.001), in favor of improvements after a VR intervention. When categorizing effect sizes, the overall effect sizes were reported as follows: 1.60 (95% CI 0.83-2.36; *z*=4.09; *P*<.001) for the effect of VR on pain (n=31), 1.40 (95% CI 0.13-2.67; *z*=2.17; *P*=.03) for functioning (n=60), 0.49 (95% CI −0.71 to 1.68; *z*=0.80; *P*=.42) for mobility (n=24), and 0.34 (95% CI −1.52 to 2.20; *z*=0.36; *P*=.72) for functional capacity (n=21).

**Conclusions:**

This systematic review revealed a broad range of outcome variables influenced by an intervention of VR technology, with statistically significant pain relief and improvements in functioning. These findings indicate that VR not only has applications in acute pain management but also in chronic pain settings, whereby VR might be able to become a promising first-line intervention as complementary therapy for patients with chronic pain.

**Trial Registration:**

PROSPERO International Prospective Register of Systematic Reviews CRD42021227016; https://www.crd.york.ac.uk/prospero/display_record.php?RecordID=227016

## Introduction

### Background

After the recent revision of the definition of *pain* in 2020 by the International Association for the Study of Pain, *chronic primary pain* is defined as pain in 1 or more anatomical regions that (1) persists or recurs for >3 months and (2) is associated with significant emotional distress (eg, anxiety, anger, frustration, or depressed mood) or significant functional disability (interference in activities of daily life and participation in social roles), and (3) the symptoms are not better accounted for by another diagnosis [1]. Approximately 19% of adults complain of chronic pain [2], resulting in significant physical and mental burden as well as significant economic and social consequences [3,4]. Pain management for chronic pain conditions that are incorporated within chronic primary pain often entails a multidisciplinary or interdisciplinary pain management program relying on a biopsychosocial approach [5], mostly with a focus on functional restoration [6]. For the management of long-term pain, public opinion is currently in strong favor of self-management strategies as a first-line effective strategy to engage patients in actively managing their own health status [7-9]. The safety and cost-effectiveness of self-management programs have been proven; nevertheless, the effect sizes are small and not sustained in the long term [7,10]. In addition, the limited efficacy of pharmacotherapy in treating chronic pain and the long-term side effects of these pharmacological treatment options [11,12] have put a premium on novel nonpharmacological therapy options for chronic pain, thereby creating room for promising new pain management strategies such as virtual reality (VR) [13,14].

VR is characterized by an artificial computer-generated environment created to replace real-world sensory inputs [15]. This technology has evolved rapidly over the last 2 decades [16] and can be operationally described as simulations that use a combination of interaction devices and sensory display systems [17]. Within VR applications, an important distinction can be made between immersive and nonimmersive media, whereby they differ based on the participant’s point of view and the experience that is produced during the application (ie, difference in spatial presence [18]) [19]. With immersive technology, participants view the full panorama, which enables the creation of a high sense of presence and immersion as if the participant is essentially inside the created environment [19]. In a nonimmersive environment, virtual content is based on how the device (PC, smartphone, or tablet) is moved or rotated, and participants are only external observers [20].

In both acute and chronic pain settings, the main idea of VR is to create distraction from the painful region [21]. VR can affect pain perception through immersive virtual environments, by occupying finite attentional resources, and by blocking external stimulation associated with the real environment and painful stimuli [22]. As distraction interventions work by competing for attention otherwise directed toward painful stimuli, pain tolerance and pain thresholds have been shown to increase under VR conditions [23]. Moreover, pain intensity, anxiety, and time spent thinking about pain decreased after VR distraction [24]. VR is thought to be more effective than traditional methods of distraction (eg, pleasant imagining, rhythmic cognitive activities, external focus of attention, and neutral imagining [25]) because of its immersive property, encompassing a patient’s visual and auditory processing and even physical actions, which, in theory, demand more attention [26]. In addition to distraction as an underlying analgesic effect of VR, the long-term use of VR is expected to induce neuroplastic changes in the sensory and motor brain regions [27].

### Objectives

Several systematic reviews have stressed the effectiveness of VR in the management of acute pain associated with medical procedures, wound debridement, and experimental pain [28-31]. The implementation of VR in chronic pain settings is still in its infancy compared with the widely accepted use of VR in acute pain settings [16]. Within the context of chronic pain, VR could be applied as an analgesic intervention and distraction method or could address pain-related behaviors [32]. Nevertheless, there is no consensus yet on which outcome measures VR has a positive effect in chronic pain settings. Therefore, this systematic review and meta-analysis aimed to evaluate the effect of VR on several outcome parameters related to the application of VR in patients with chronic pain.

## Methods

### Protocol and Registration

The study protocol was registered prospectively with PROSPERO (CRD42021227016). This systematic review and meta-analysis was conducted according to the PRISMA (Preferred Reporting Items for Systematic Reviews and Meta-Analyses) guidelines [33].

### Search Strategy

A search strategy based on the PICO (Patient or Population, Intervention, Comparison, and Outcome; evidence-based search strategy focusing on patients or populations, interventions, comparisons, and outcomes) framework was developed [34]. The following research question was constructed: *The effects of virtual reality (intervention) on multiple outcome measurements (outcome) in patients with chronic pain (population).* The component *control* was not defined for this question. The final search strategy was built by combining free and Medical Subject Headings terms. Within *Population*, *Intervention*, and *Outcome*, search terms were combined using the Boolean term OR. The Boolean term AND was used between the complete search terms for *Population*, *Intervention*, and *Outcome*. The search strategy was conducted using the following databases: PubMed, Embase, Scopus, and Web of Science from their outset until December 2020. No limitations were applied to the search. The full search strategy for PubMed is outlined in Multimedia Appendix 1.

### Eligibility Criteria

Studies exploring the effects of VR in patients with chronic pain were eligible for inclusion. Studies with both adults and children were eligible if the study participants experienced chronic pain. Chronic pain was defined as pain that lasted for >3 months [35], including patients with primary and secondary chronic pain conditions. Studies in which the effect of VR was explored in healthy participants or in acute pain settings were excluded. For this intervention, there were no restrictions on the VR devices, and all types of VR were permitted, including (but not limited to) studies with head-mounted displays, video games, displays with body motion sensors, Nintendo Wii consoles, Xbox, PlayStation, and computers. Studies that did not use VR to explore its effects on patients with chronic pain were excluded. There were no limitations in the outcome measurements because the goal was to explore the effects of VR on all types of outcome variables that were recorded in this setting. Studies reporting in languages other than English, Dutch, French, or German were excluded. Publications available only in abstract form, conference abstracts, expert opinions, letters to the editor, study protocols, reviews, meta-analyses, or meeting reports were considered not suitable for inclusion.

### Study Selection

After deduplication using the EndNote X9 reference manager (Clarivate), 2 reviewers (JJ and VN) independently screened all retrieved articles for titles and abstracts using the Rayyan web-based software (Rayyan Systems Inc) [36]. Subsequently, the same 2 reviewers performed the full-text screening (independently). If discrepancies occurred, consensus was sought through consultation and discussion with a third independent reviewer (ADS).

### Data Extraction

All relevant information concerning the possible effects of VR in patients with chronic pain was synthesized in an a priori constructed evidence table. The following items were extracted from each of the remaining articles: author, publication year, country, study design, sample size (including sex distribution), underlying pathology, VR application (including duration, type of VR, and device), and reported outcome measurements. Data extraction was performed by one reviewer (JJ) and checked for correctness by another (LG). Any discrepancies were discussed in a consensus meeting with all the reviewers.

### Risk of Bias Assessment

A modified version of the Downs and Black checklist was used to assess the quality of the included studies [37]. This instrument, consisting of 27 items, was developed to evaluate the methodological quality of several study designs, including randomized trials, nonrandomized trials, and observational studies [37]. All items were categorized into 5 subscales: reporting, external validity, bias, confounding, and power. Each item was given a score of 0 (*no*) or 1 (*yes*), except for item 5, where a score of 1 meant the item was partially presented and a score of 2 if a complete description was presented. The answer option *not applicable* was also available. The scoring of item 27, which refers to the power of the study, had been modified in the sense that it received a score of 1 if a power calculation was performed and 0 otherwise [38,39]. After scoring each individual item, all included studies were categorized as having poor, fair, good, or excellent quality based on the total score for further data synthesis. A total score of ≤14 out of 28 was considered poor quality, 15-19 was considered fair, 20-25 was considered good, and 26-28 was considered excellent methodological quality [40].

### Statistical Procedure for the Meta-analysis

The standardized mean difference was chosen as the effect size to compare the differences between measurements before and after the VR intervention, calculated as the difference in sample means after VR minus before VR, divided by the SD before VR (g gain).nOnly sample SDs of the measurements before VR, instead of pooled SDs, were used in the calculation because they are not influenced by VR effects and are therefore more likely to be consistent across studies [41]. Correlations between measurements were estimated to be 0.5. As standardized mean difference does not correct for differences in the direction of the scale, the mean values from outcomes that have a higher score when they reveal an improvement are multiplied by −1 to ensure that all the scales point in the same direction. Thus, the effect size measures are positive if the data indicate a desirable effect of VR. Several studies included more than one outcome measurement, wherefore a random-effect 3-level meta-analysis was performed [42]. Thus, a 3-level meta-analysis model was fitted with sampling variance at the first level, within-study variance at the second level, and between-study variance at the third level [42]. Meta-analyses were performed for all effect sizes combined and for effect sizes categorized according to the type of outcome measurements (ie, pain, functioning, mobility, and functional capacity). Meta-analyses were performed using R Studio (R Foundation for Statistical Computing) version 1.4.1106 (R version 4.0). *P*≤.05 was considered statistically significant. Figures were created based on the code provided by Fernández-Castilla et al [43] to visually present the results of meta-analyses of multiple outcomes. Only studies with sufficient data were included in this meta-analysis. If insufficient information was provided, the authors were contacted to provide more in-depth data.

## Results

### Study Selection

**Figure 1:**
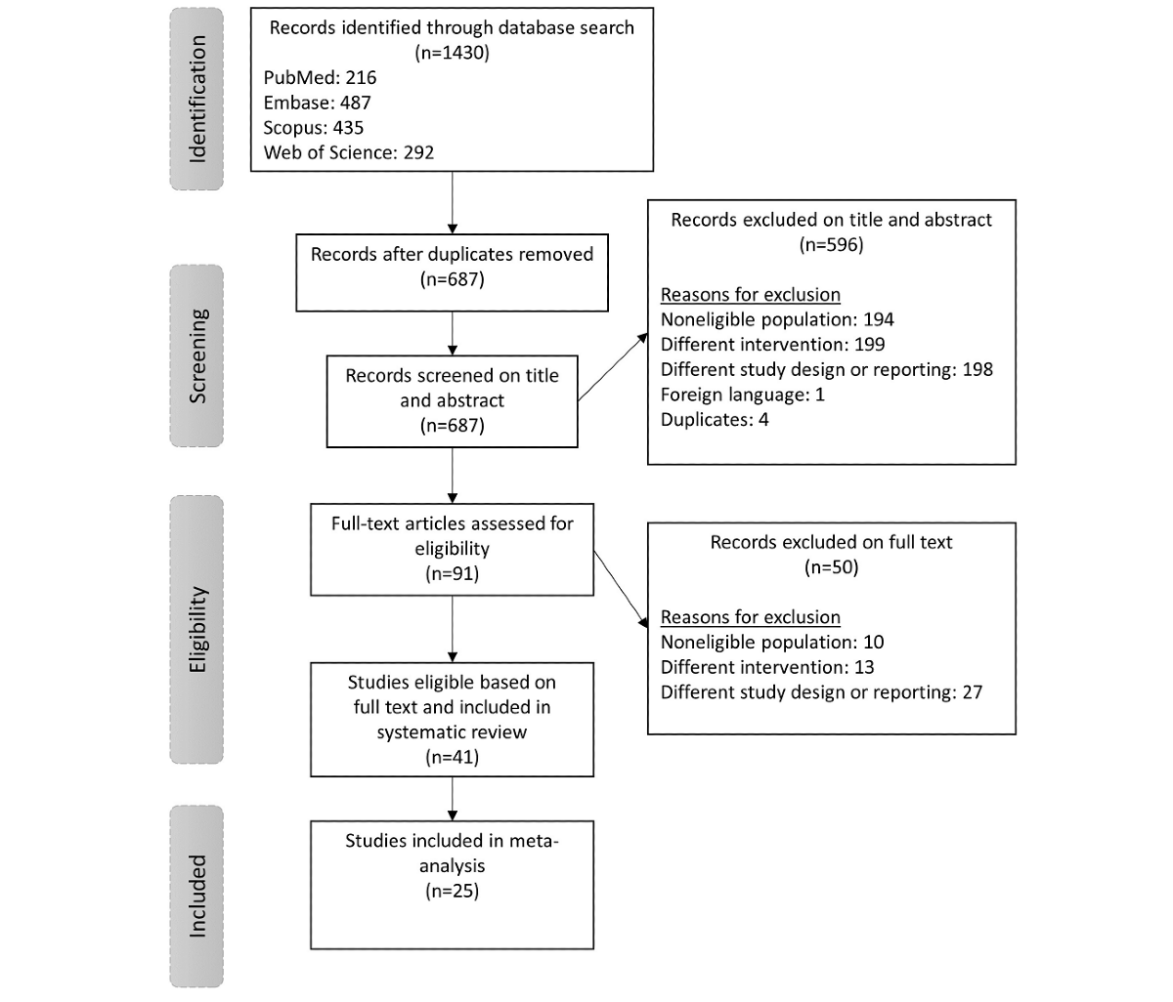
PRISMA (Preferred Reporting Items for Systematic Reviews and Meta-Analyses) flowchart.

A total of 1430 articles were identified through 4 database searches (Figure 1). After removing duplicates, 687 (48.04%) articles were selected for screening. After screening titles and abstracts, 13.2% (91/687) of articles remained eligible for full-text screening. Studies were excluded based on study design or reporting (n=198), no patients with chronic pain (n=194), no VR as intervention (n=199), duplicates (n=4), or other languages (n=1). The percentage of agreement on title and abstract screening between both reviewers was 92.8%. After full-text screening, 41 articles were included in this systematic review. The primary reasons for study exclusion were no patients with chronic pain (n=10), no VR as intervention (n=13), and a different study design or reporting (n=27). The percentage agreement between both reviewers for full-text screening was 95.7%. Data from 25 studies were included in the meta-analysis.

### Study Characteristics

A comprehensive overview of the characteristics of the included studies is presented in Table 1. The earliest study included in this review was published in 2008 and the most recent study, in 2020. In terms of study design, the following studies were included: 18 quasi-experimental study designs, 16 randomized controlled trials, and 7 case series.

In terms of population, most studies (n=11) investigated adults with chronic pain without further specification of the specific type of chronic pain [44-54]. A total of 7 studies evaluated the effect of VR in patients with fibromyalgia [55-61], 6 studied patients with chronic low back pain [62-67], 5 studied patients with chronic neck pain [68-72], 4 studies evaluated VR in patients with (upper limb) complex regional pain syndrome [73-76], and 3 studied patients with phantom limb pain [77-79]. In addition, 1 study investigated pediatric patients with chronic pain [80], and 1 study investigated both adults and adolescents [73]. A total of 1232 patients were enrolled in 41 studies, of which 784 (63.64%) were women.

With regard to the VR application, the duration of the sessions ranged from 2×2 minutes with 30 seconds rest between sessions [64] up to sessions of 1 hour [60]. The frequency varied from 1 session [64] to 2 sessions per week for 24 weeks [60].

**Table 1:** Characteristics of the included studies.

Authors	Country	Design	Participants, N (number of female participants)	Population	Duration of the intervention	VR^a^ device and application	Type of VR	Outcome measurements
Alemanno et al [62]	Italy	QE^b^	20 (11)	Adults with chronic low back pain	1 hour per session for 12 sessions over 4-6 weeks	VR Rehabilitation System, Khymeia, Italy	Nonimmersive	Roland and Morris Disability Questionnaire, Repetition Index, SF-36 Short Form Health Survey, NRS^c^ Pain, McGill Pain Questionnaire, Brief Pain Inventory, Beck Depression Inventory, and neuropsychological evaluations
Amin et al [44]	Canada	RCT^d^	30 (13)	Adult patients with chronic pain	2×10 minutes	Oculus Rift DK2 and a Cardboard VR to play InMind	Immersive	Present and retroactive pain intensity
Botella et al [55]	Spain	QE	6 (6)	Adult patients with fibromyalgia	7 weeks with ten 2-hour sessions: sessions 1 through 6 were delivered twice a week for 3 weeks, whereas sessions 7 through 10 were delivered weekly for 4 weeks	2 PCs, a large projection screen, 2 projectors, a wireless pad, and a speaker system. Application used: EMMA^e^ World	Nonimmersive	Beck Depression Inventory II, Positive and Negative Affect Schedule, Chronic Pain Coping Inventory, Fibromyalgia Impact Questionnaire, and VR Satisfaction Scale
Brown et al [63]	United States	RCT	45 (27)	Adults with chronic low back pain, receiving spinal injections	One 5-minute session	VR immersive format in the Oculus GoÒ headset	Immersive	Anxiety thermometer, NRS pain, Patient-Reported Outcomes Measurement Information System, Modified Oswestry Disability Index, and VR Symptom Questionnaire
Chau et al [73]	United States	CS^f^	8 (7)	Upper limb complex regional pain syndrome (adults and 1 adolescent)	10 sessions (1-3 per week) with 45-60 minutes each session	HTC Vive VR System (virtual 3D kitchen environment)	Immersive	Short Form McGill Pain Questionnaire, VAS^g^, Wong-Baker Faces Scale, and Subjective feedback
Collado-Mateo et al [56]	Spain	RCT	83 (83)	Adult patients with fibromyalgia	Twice a week for 1 hour per session over 8 weeks	An exergame called VirtualEx-FM based on Microsoft Kinect	Nonimmersive	Timed up and go test, functional reach, Clinical Test of Sensory Integration of Balance, fear of falling using VAS
Darnall et al [81]	United States	RCT	88 (ND^h^)	Adults with chronic nonmalignant low back pain or fibromyalgia	21 days	Oculus Go VR Headset (visual biofeedback in relaxation training)	Immersive	Defense and Veterans Pain Rating Scale; pain interference on activity, mood, sleep, and stress; pain catastrophizing scale; pain self-efficacy; global impression of change; satisfaction with treatment; and motion sickness and nausea
Fowler et al [45]	United States	QE	16 (3)	Veterans with chronic pain	19-day program with 20 minutes per VR session	Oculus Rift and Samsung Oculus Gear VR	Immersive	VR feasibility, Pain Outcomes Questionnaire-VA, Fear of Daily Activities, questionnaire for kinesiophobia, Pain Catastrophizing Scale, and patient-specific functional scale
Garcia-Palacios et al [57]	Spain	RCT	61 (61)	Adults with fibromyalgia	Six 2-hour group sessions delivered twice a week	2 PC computers with an EMMA VR environment	Nonimmersive	Fibromyalgia Impact Questionnaire, Brief Pain Inventory, Chronic Pain Coping Inventory, Beck Depression Inventory II, Quality of Life Index, and acceptability and satisfaction
Garrett et al [46]	Canada	CS	8 (6)	Adults with chronic pain conditions	1 month with 12 sessions of 30 minutes each	Oculus Rift DK2 1100 field of view stereoscopic HMD^i^	Immersive	NRS pain, Brief Pain Inventory, Self-Administered Leeds Assessment of Neuropathic Symptoms and Signs, cybersickness, and individual interviews
Griffin et al [80]	United States	QE	17 (13)	Pediatric patients with chronic pain	1-8 sessions of 30 minutes (once a week)	HTC Vive VR system	Immersive	Presence, child daily questionnaire, and interviews
Gromala et al [47]	Canada	RCT	13 (7)	Adult patients with chronic pain	1 moment	Technology’s DeepStream VR with virtual meditative walk	Immersive	NRS pain
Guarino et al [48]	Italy	CS	11 (8)	Adult patients with chronic pain	8 VR sessions of 30 minutes, 2 times a week	VR scenarios were run in a PC, and the environments were visualized on a monitor	Nonimmersive	McGill Pain Questionnaire, Brief Pain Inventory Severity and Interference, State trait Anxiety Inventory, Beck Depression Inventory, VAS, and subjective units of distress scales
Harvie et al [68]	Australia	CS	12 (3)	Adults with chronic neck pain	36-70 days with 10 minutes of VR twice a day	Samsung Gear VR system with Motor Offset Visual Illusion	Immersive	Pain threshold and NRS pain
Herrero et al [58]	Spain	QE	40 (40)	Adults with fibromyalgia	3 sessions of 20 minutes each	2 PC computers, a 3.4-m screen made of reflective material, 2 projectors, and a Dolby 7.1 surround sound audio system with EMMA	Nonimmersive	Mood State, NRS pain, NRS fatigue, NRS motivation, NRS self-efficacy, and NRS of several emotions (joy, sadness, anger, surprise, anxiety, calmness, and vigor/energy)
House et al [82]	United States	QE	12 (12)	Adults with persistent pain in shoulder and arm following postsurgical breast cancer	20-50 minutes twice week for 8 weeks	BrightArm Duo technology with 3D custom integrative rehabilitation games	Nonimmersive	NRS pain, upper limb range of motion, Beck Depression Inventory II, Neurophysiological Assessment Battery, Revised Hopkins Verbal Learning Test, Revised Brief Visuospatial Memory Test, and Trail Making Test
Jin et al [49]	Canada	RCT	20 (16)	Adults with chronic pain	1 moment, 35-45 minutes per participant with 10-minute VR	Oculus Rift DK2 with Cryoslide game	Immersive	Pain intensity (VAS) and distraction
Jones et al [50]	United States	QE	30 (20)	Adults with chronic pain conditions	Single 5-minute exposure to VR	Oculus rift DK2 and Deepstream VR with cool!	Immersive	NRS pain, engagement, and side effects
Matamala-Gomez et al [74]	Spain	QE	19 (14)	Adults with neuropathic chronic pain in the upper limb	Single session of 55 minutes	HMD rift development kit 2, Oculus	Immersive	Ownership, agency, mental representation, NRS pain, and VR questionnaire
Matheve et al [64]	Belgium	RCT	84 (54)	Adults with chronic low back pain	2×2 minutes with 30 seconds of rest in between	Valedo Pro, Hocom	Nonimmersive	NRS pain, Roland and Morris Disability Questionnaire, Pain Catastrophizing Scale, Tampa Scale for Kinesiophobia, pain intensity, time spent thinking about pain, and pelvic tilts
Monteiro et al [65]	Portugal	RCT	34 (34)	Adults with chronic low back pain	8 weeks with sessions 3 times a week for 90 minutes	Nintendo Wii motion and Wii balance board	Nonimmersive	NRS pain, balance, sit-to-stand test, and profile of mood states
Mortensen et al [59]	Denmark	QE	15 (15)	Adults with fibromyalgia	15 sessions of 30 minutes	Motion-controlled video games, Wii, Ps3, and Xbox Kinect	Nonimmersive	Pain VAS, Brief Fatigue Inventory, Activities of Daily Life Questionnaire, test of playfulness, and interviews
Mouraux et al [76]	United States and Belgium	QE	22 (12)	Adults with chronic neuropathic pain in unilateral upper extremity	5 sessions of 20 minutes over 1 week	3D augmented reality system with a 3D display (Kit Nvidia 3D Vision) and 3D camera (Xbox 360 Kinect)	Nonimmersive	Pain VAS, McGill Pain Questionnaire, and Douleur Neuropathique 4 Questions
Ortiz-Catalan et al [77]	Sweden and Slovenia	QE	14 (ND)	Adults with chronic intractable phantom limb pain	Twice per week, 12 sessions of 2 hours each	Neuromotus and Integrum AB	Nonimmersive	NRS intensity, frequency, duration, and quality of phantom limb pain; Pain Rating Index Scale; Short form of McGill questionnaire; and interviews
Pamment and Aspell [51]	United Kingdom	QE	18 (12)	Adults with chronic pain	4 conditions, each lasting 2 minutes	HMD (WRAP 1200, Vuzix) connected to a video camera	Immersive	McGill Pain Questionnaire and illusion and control questions
Phoon Nguyen et al [83]	Australia	QE	9 (7)	Adults with burning mouth syndrome	1 session of 3 experimental conditions of 1 minute each	MIRAGE-mediated reality system; computer screen displaying live digitally manipulated video feed of their own face	Nonimmersive	Pain VAS, Wong-Baker Faces Pain Rating Scale, and VAS burning pain/sensation
Rezaei et al [69]	Iran	RCT	44 (ND)	Adults with chronic neck pain	8 training sessions over 4 weeks with 21 minutes each	Cervigame head mouse extreme	Nonimmersive	Pain VAS, Neck Disability Index, and Y-balance test
Rutledge et al [78]	United States	QE	14 (1)	Adults with phantom limb pain	57 treatment sessions with 40-60 minutes per session	VR treatment based on mirror therapy with an Oculus Rift Headset	Immersive	Phantom Limb Pain Questionnaire, Trinity Amputation and Prosthetic Experience Scale-Short Form-12, Patient Health Questionnaire-9, Posttraumatic Stress Disorder Checklist-Military version, and Present Questionnaire
Sarig-Bahat et al [70]	Australia	RCT	32 (22)	Adults with chronic neck pain	4-6 supervised intervention sessions for 30 minutes each over a period of 5 weeks+home training sessions of 30 minutes for at least three times a week	HMD with a Wrap 1200VR by Vuzix	Immersive	VAS pain, Neck Disability Index, Tampa Scale for Kinesiophobia, static and functional balance, satisfaction, global perceived effect, and range of motion
Sarig-Bahat et al [71]	Australia and Israel	RCT	90 (63)	Adults with chronic neck pain	5 minutes, 4 times a day, 4 days per week, for 4 weeks	Oculus Rift DK1 HMD with 3D motion tracking	Immersive	Neck disability Index, global perceived effect, VAS pain, self-rated health status in the European life quality questionnaire, velocity, Tampa scale for kinesiophobia
Sato et al [75]	Japan	CS	5 (4)	Adults with complex regional pain syndrome	5-8 weeks with 1 session each week	FASTRAK and cyberglove, PC desktop with a CyberGlove as hand input, FASTRAK as real-time position and motion tracker, and computer screen	Nonimmersive	VAS pain and range of motion
Shahrbanian et al [52]	Canada	QE	12 (5)	Adult stroke patients with chronic pain	1 moment with 3-5 minutes of each VR condition	HMD Kaiser Optical System with Nvidia Quadro FX 4500 graphics card	Immersive	Pain threshold, engagement, VAS mood, and NRS pain
Solca et al [84]	Switzerland	QE	15 (5)	Adults with chronic leg pain with a spinal cord implant	1 moment	Oculus Rift CV1 with RealiSM software	Immersive	Analgesia and embodiment
Tejera et al [72]	Spain	RCT	44 (23)	Adults with nonspecific chronic neck pain	2 sessions per week for 4 weeks	VR Vox Play glasses used with an HMD clamping system	Immersive	VAS pain, pain pressure threshold, temporal summation, range of motion, neck disability index, pain catastrophizing scale, Tampa scale for kinesiophobia, fear-avoidance beliefs questionnaire, and Pain Anxiety Symptoms Scale
Thomas et al [66]	United States	RCT	53 (ND)	Adults with chronic low back pain	3 consecutive days	Samsung 3D shutter glasses with Vizard software	Nonimmersive	Changes in lumbar spine flexion, VAS expectations of pain and harm, and game experience survey
Tong et al [79]	China	CS	5 (0)	Adults with phantom limb pain	10 sessions over 6 weeks	Immersive room-scale VR system and HMD from HTC Vive with Unity 3D	Immersive	Short Form McGill Pain Questionnaire, VAS pain, NRS embodiment, NRS ownership, and hospital anxiety and depression scale
Trujillo et al [67]	United States	CS	2 (0)	Adults with chronic low back pain	7 sessions with 2 sessions per week of 30-45 minutes each	Virtual embodiment training with Virtual Embodiment Training (KVET); HTC Vive with a VR HMD	Immersive	Simulator Sickness Questionnaire; VAS pain and Pain Catastrophizing Scale
Villafaina et al [60]	Spain	RCT	55 (55)	Adults with fibromyalgia	2 sessions of 1 hour per week for 24 weeks	VirtualEx-FM	Nonimmersive	Chair-stand test, 10-step stair test, 6-minute walk test, Fibromyalgia impact questionnaire, and International Physical Activity Questionnaire
Villafaina et al [61]	Spain	RCT	55 (55)	Adults with fibromyalgia	2 sessions of 1 hour per week for 24 weeks	VirtualEx-FM	Nonimmersive	Electroencephalography
Wiederhold et al [53]	United States	QE	31 (ND)	Adults with chronic pain	5-minute pain focus session followed by a 20-minute intervention session	Mobile phone VR therapy and an HMD	Immersive	Simple Descriptive Pain Intensity Scale, Numerical Pain Intensity Scale, VAS pain, and physiological measures (heart rate, peripheral skin temperature, respiration, and skin conductance)
Wiederhold et al [54]	Belgium and United States	QE	40 (ND)	Adults with chronic pain	15-minute exposure session	VR exposure while wearing HMD	Immersive	Pain reduction, pain focus, and skin temperature

^a^VR: virtual reality.

^b^QE: quasi-experimental.

^c^NRS: Numeric Rating Scale.

^d^RCT: randomized controlled trial.

^e^EMMA: Engaging Media for Mental Health Applications.

^f^CS: case series.

^g^VAS: visual analog scale.

^h^ND: not displayed.

^i^HMD: head-mounted display.

### Risk of Bias

The range of the total scores on the Downs and Black checklist varied between 8 out of 28 and 25 out of 28. Overall, 5 (12%) of the 41 included studies scored poor on the risk of bias assessment (total score ≤14), 17 (42%) had a fair score (total score between 15 and 19), and 19 (46%) had good quality (total score between 20 and 25). The total scores on the risk of bias assessment are presented in Table 2. Multimedia Appendix 2 presents the full results of the risk of bias assessment.

In the 18 studies with a quasi-experimental design, low scores were found for the external validity subscale. Only 6 (33%) of the 18 studies had an accurate score on the item of whether patients were representative of the entire population from which they were recruited, and the item concerning representativeness of staff, places, and facilities where the patients were treated was only considered representative of the treatment most patients received in 7 (39%) of 18 studies. Several items that evaluated internal validity performed poorly, including the attempt to blind study participants, which was only evaluated efficiently in 1 (6%) of the 18 studies. No study has attempted to blind outcome assessors. Recruiting patients over the same period was only scored satisfactory in 3 (17%) studies, no studies randomized patients, and adjustments for confounding were only performed in 6 (33%) of 18 studies.

In the 16 randomized controlled trials, several items evaluating internal validity received low scores. Of the 16 studies, only 1 (6%) study provided information on attempts to blind study participants, whereas 5 (31%) studies provided information on attempts to blind outcome assessors. Information regarding adjustments for confounding was provided in only 7 (44%) studies.

A total of 7 case series were included; only 2 (29%) studies reported the estimates of random variability. For the 3 items evaluating external validity, most studies performed poorly with representativeness ranging from 14% (1/7) to 43% (3/7). Of the 7 studies, on the subscale for internal validity (bias), only 1 (14%) study attempted to blind the study participants, and none attempted to blind the outcome assessor. Patients were not recruited in the same period within a study, and only 1 (14%) of 7 studies used randomization (no concealed randomization). Adjustments for confounding were explored in 2 (29%) studies, and a power calculation was reported in 3 (43%) of the 7 studies.

**Table 2:** Total score on the risk of bias assessment.

Study^a^	Total score (out of 28)	Category
Alemanno et al [62]	22	Good
Amin et al [44]	19	Fair
Botella et al [55]	16	Fair
Brown et al [63]	24	Good
Chau et al [73]	16	Fair
Collado-Mateo et al [56]	24	Good
Darnall et al [81]	25	Good
Fowler et al [45]	21	Good
Garcia-Palacios et al [57]	23	Good
Garrett et al [46]	18	Fair
Griffin et al [80]	20	Good
Gromala et al [47]	18	Fair
Guarino et al [48]	10	Poor
Harvie et al [68]	20	Good
Herrero et al [58]	18	Fair
House et al [82]	19	Fair
Jin et al [49]	18	Fair
Jones et al [50]	16	Fair
Matamala-Gomez et al [74]	19	Fair
Matheve et al [64]	24	Good
Monteiro et al [65]	21	Good
Mortensen et al [59]	18	Fair
Mouraux et al [76]	14	Poor
Ortiz-Catalan et al [77]	18	Fair
Pamment and Aspell [51]	20	Good
Phoon Nguyen et al [83]	21	Good
Rezaei et al [69]	25	Good
Rutledge et al [78]	18	Fair
Sarig-Bahat et al [70]	23	Good
Sarig-Bahat et al [71]	21	Good
Sato et al [75]	12	Poor
Shahrbanian et al [52]	17	Fair
Solca et al [84]	19	Fair
Tejera et al [72]	23	Good
Thomas et al [66]	25	Good
Tong et al [79]	16	Fair
Trujillo et al [67]	17	Fair
Villafaina et al [60]	23	Good
Villafaina et al [61]	22	Good
Wiederhold et al [53]	11	Poor
Wiederhold et al [54]	8	Poor

^a^Each study was scored on all 27 items of the modified Downs and Black checklist. On the basis of the total score, all included studies were categorized as presenting poor, fair, good, or excellent quality. A total score of ≤14 out of 28 was considered poor quality, 15-19 was considered fair, 20-25 was considered good, and 26-28 was considered excellent methodological quality.

### Interventions

A total of 23 studies used immersive VR techniques, and 18 used non-immersive techniques. Within the category of immersive techniques, VR games (7/23, 30%), mindfulness-based interventions (6/23, 28%), practical exercises (6/23, 28%), and visual illusions (4/23, 17%) were used. In the category of nonimmersive techniques, of the 18 studies, 6 (33%) used exergames, 3 (17%) used an avatar or exoskeleton, and 9 (50%) studies used a television or PC screen. A more detailed description of immersive techniques is provided in further sections, followed by an in-depth explanation of nonimmersive VR techniques.

When VR was applied as a game, a broad range of VR games were used, including shooter games [44], a game of grasping where participants had to stomp fruit [80], a game with sliding in an icy cave during which participants should hit creatures with snowballs [49], a game in which the user travels through a landscape with interaction [50], a game with a visualization of a red airplane that could be controlled by head motion and the user could hit targets [70,71], and a game of pushing a ball of the table and shooting a basketball [79].

When VR was used in the context of mindfulness-based or relaxation treatments, several applications were used, including a 5-minute relaxation video [63], sessions to support patients in learning self-management skills based on cognitive behavior therapy principles in which the VR headset was used for visual biofeedback in relaxation training [81], exploratory environments [46,47], interaction with simulation graphics and exploration of virtual worlds [53], and relaxing environments and sounds [54].

In addition, immersive VR was also used to practice exercises in a kitchen environment in which participants had to perform tasks representative of daily activities [73]. In addition, minimal exercises such as neck and head movements and larger exercises such as torso and upper extremity movements were provided [45,68]. Participants could bicycle on a pedaller through VR environments [78], practice neck exercises through the illusion of diving with sounds of the sea [72], and perform exercises based on the principles of graded motor imagery to relearn associations to pain and improve their function [67].

Finally, other possibilities of immersive VR were seeing an illusion of the affected body part through an avatar [74], a visualization of their own back and synchronously or asynchronously tapping with a wooden stick [51], experiencing hot and cold stimuli through a snow world environment and a canyon environment, respectively [52], and a visual illumination of a circumscribed skin region in the VR corresponding with Spinal Cord Stimulation [84].

In contrast to immersive applications, several studies have used nonimmersive VR techniques. The studies (n=6) that made use of exergames consisted of a variety of exercises, such as aerobic sessions; postural control and coordination; exercises to improve mobility skills, fitness, ability, and balance [56,60,61]; Wii Fit Plus workouts [65]; a sports game package [59]; and an exercise in which a real-time 3D image of the individual’s moving, nonaffected body part was presented, and participants played a game to touch a few targets with the hand or fingers of the virtual affected upper extremity [76].

An avatar or exoskeleton was used in 3 studies as virtual rehabilitation and could help to teach patients to execute correct movements with the painful body parts to regain a correct body image [62] or with games for unimanual and bimanual motor, emotive, and cognitive training [82] or with pelvic tilt exercises where the VR was used as a game [64].

A television or PC screen was used in the following situations (n=9): support of a group cognitive behavior therapy containing speciﬁc content for developing relaxation and mindfulness skills, as an adjunct to the activity pacing component, with education, activity management, and relapse prevention, to induce positive emotions and promote motivation, self-efficacy, and behavior activation [48,55,57,58]. Other authors used a television or PC screen to practice motor execution by games, such as racing cars using phantom movements and matched random target postures of a virtual arm [66,77], a rabbit attempting to reach carrots and avoid obstacles [69], or a target-oriented motor control task where hand exercises consisted of reaching out, grasping, transferring, and placing [75]. In another study, patients with burning mouth syndrome watched their tongues on a computer screen with illusions and performed tongue movements [83].

### Instruments of Outcome Measurements

Multimedia Appendix 3 provides a complete summary of the measurement instruments used to evaluate these outcomes. Of the 41 included studies, 35 (85%) evaluated pain-related outcomes [44-54,57-59,62-73,75-81,83,84]. Psychological outcomes, such as kinesiophobia and fear, mood, satisfaction, expectations of pain, pain focus, time spent thinking about pain, self-efficacy, emotions, motivation, stress, catastrophizing, acceptability, global impression of change, ownership, and agency, were measured in 19 studies [45,48,52,55-59,62-65,67,70-72,74,81,82]. Functional outcomes, including disability, physical comfort, strength, fitness, and sleep, were measured in 15 studies [45,55-57,59,60,62,64,65,69-72,77,82]. Functional capacity (evaluated by measuring balance, repetition index, step test, and composite value) was measured in 5 studies [56,62,65,69,70]. Mobility (range of motion) was measured in 4 studies [45,62,71,72]. Neuropsychological functions were measured in 2 studies [61,82] using resting brain dynamics with electroencephalography, Brief Visuospatial Memory Test-Revised, Neurophysiological Assessment Battery, Revised Hopkins Verbal Learning Test, Trail Making Test, and other neuropsychological evaluations. Quality of life was measured in 3 studies [57,62,71]. Other sensations were measured in 5 studies [52,54,68,72,78]. The experience of VR technology, such as presence, simulator sickness, physical comfort, feasibility, safety, effort put in the game, and satisfaction, was measured in 18 of the included studies [45,46,50-52,55,57,59,63,66,67,70,74,78-81,84] using the VR Satisfaction Scale, VR Symptom Questionnaire, satisfaction with treatment (numeric rating scale), motion and nausea (numeric rating scale), test of playfulness, illusion and control questions, questionnaire of embodiment, and a game experience survey.

### Meta-analysis of Outcome Measurements

**Figure 2:**
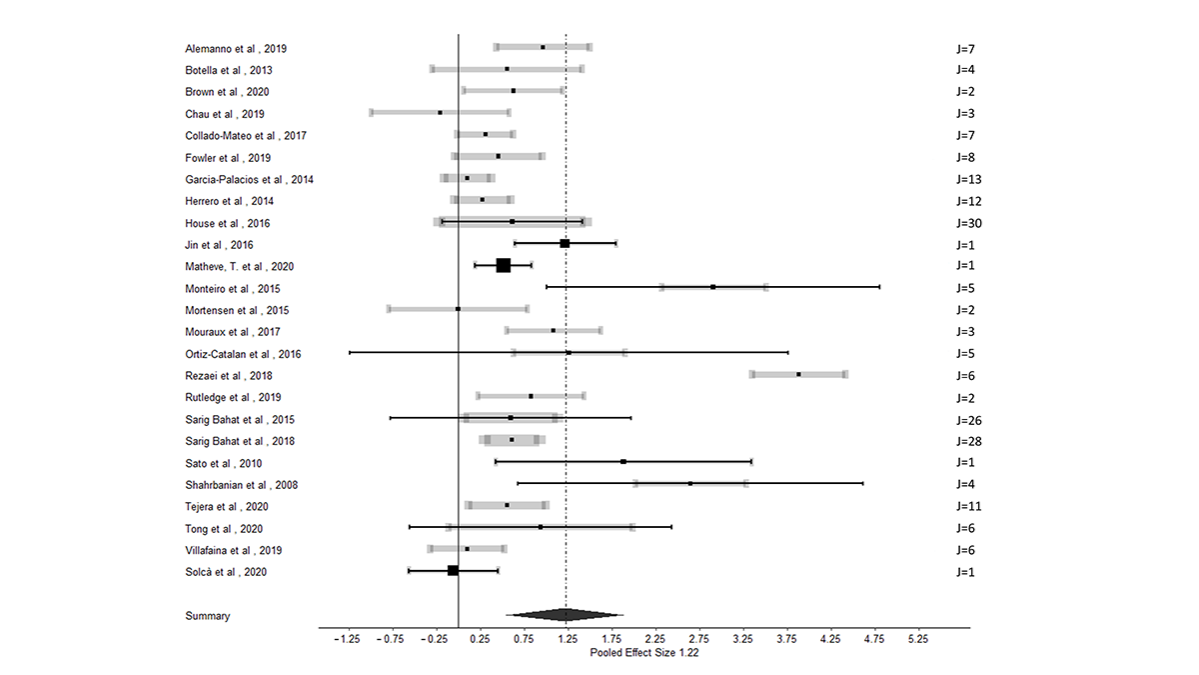
Summary forest plot for the effect of virtual reality on all outcome measurements in patients with chronic pain. Each line presents the results of 1 study (potentially including multiple effect sizes). The meta-analytic mean, with the corresponding 95% CI, is presented with a black dot and black line. This black 95% CI represents the total study precision. The additional CI in gray is based on the sampling variance of individual observed effect sizes of the study to obtain a visual contribution of the study sample size on the total study precision. The thickness of the gray CI is proportional to the number of effect sizes within studies. The number of effect sizes per study is presented as "J" on the right side of the figure [45,49,52,55-60,62-65,69-73,75-79,82,83].

**Figure 3:**
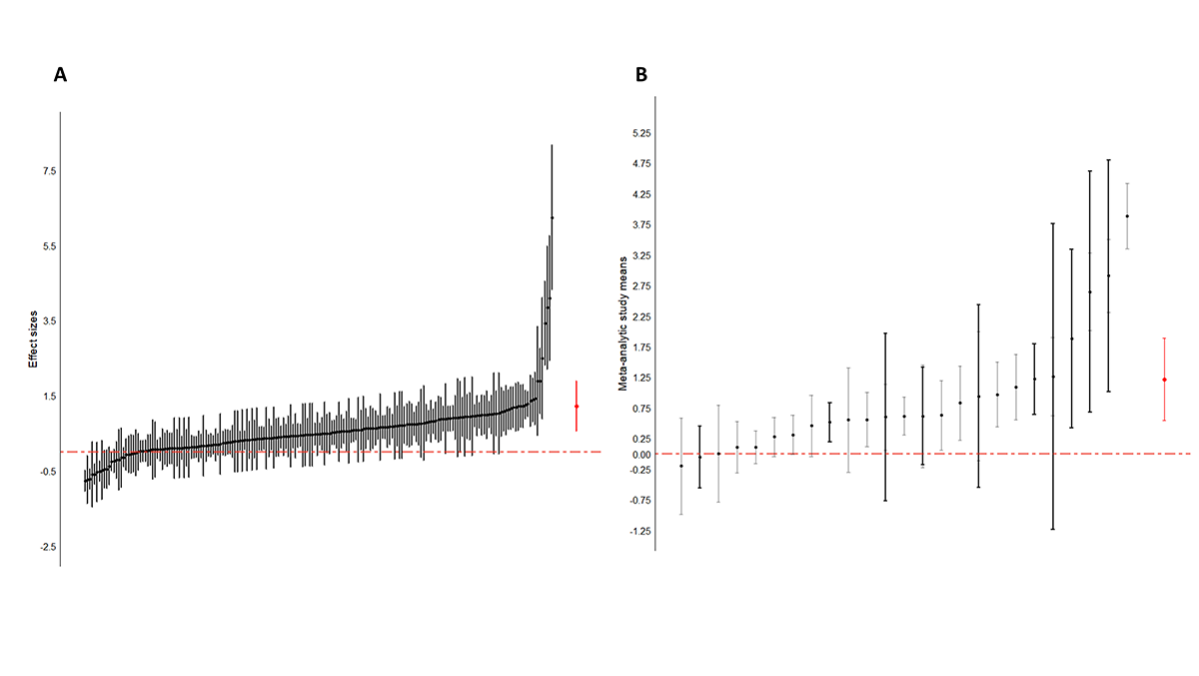
Caterpillar plots with all effect sizes (A) and study effect sizes (B). The overall effect size is presented in red.

**Figure 4:**
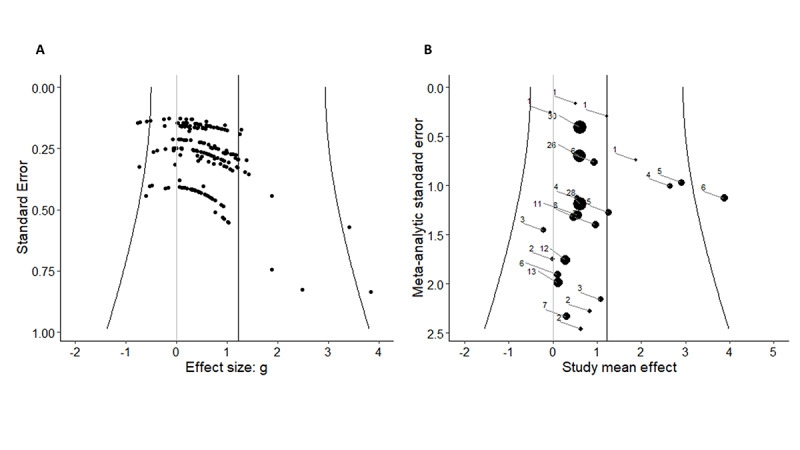
A funnel plot of all effect sizes (A) and a study funnel plot (B).

In most (21/25, 84%) studies, more than one outcome measurement was used to evaluate the effect of VR in patients with chronic pain, resulting in 194 effect sizes from 25 studies. Figure 2 [45,49,52,55-60,62-65,69-73,75-79,82,84] presents the summary forest plots, where each line represents all the outcomes from a particular study. If 0 was included in the CI, the reported effect size was not statistically significant at the 5% level. The overall effect size based on the 3‐level meta‐analysis was estimated at 1.22, with an SE of 0.34 and a 95% CI of 0.55-1.89 (*z*=3.56; *P*<.001). This indicates that VR intervention in patients with chronic pain had a positive effect on the outcome measurements used. Caterpillar plots are visualized in Figure 3 to provide a general view of the distribution of all effect sizes (Figure 3A) and study effect sizes (Figure 3B). Funnel plots are scatter plots in which effect sizes are plotted against the SE with small SEs at the top of the graph. Funnel plots for this meta-analysis are displayed in Figure 4, demonstrating an asymmetrical plot, which might be interpreted as an indication of publication bias.

The type of VR intervention (binary factor: immersive vs nonimmersive) was included as an additional covariate in the analysis; however, the type of VR intervention was not statistically significant (*F*_1,192_=0.88; *P*=.35). Given these results, it can be concluded that the overall effect of VR on several outcome measurements is not moderated by the type of VR intervention. In addition, the type of pain (primary or secondary) and the objective of VR (exercise, virtual illusion, distraction, or cognitive therapy) were added as potential moderators to the analyses, whereby the omnibus test for the moderator analysis was not statistically significant for the type of pain (*F*_1,180_=0.03; *P*=.86), nor for the objective of VR (*F*_1,190_=0.59; *P*=.62).

**Figure 5:**
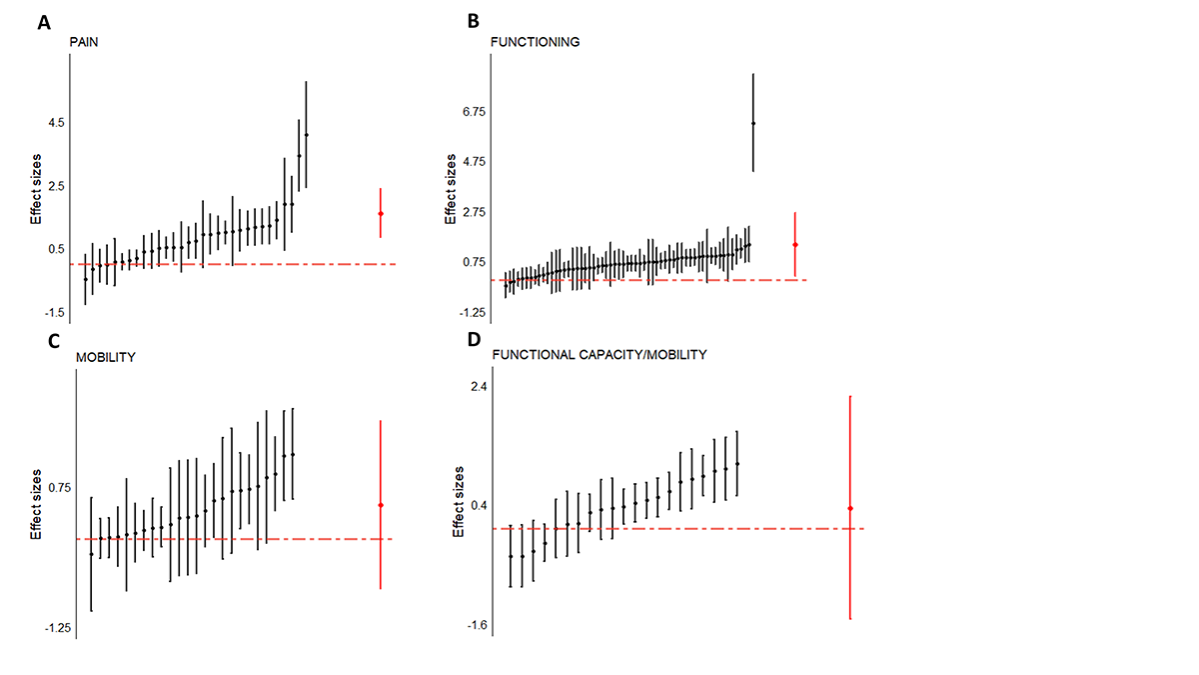
Reported effect sizes from the studies exploring (A) pain, (B) functioning, (C) mobility, and (D) functional capacity. Significant effects were found for pain and functioning outcome variables. Effect sizes from individual studies are presented in black, and the overall effect size is shown in red.

When specifically focusing on effect sizes that measure the effect of VR on pain (n=31), an overall effect size of 1.60 (SE 0.39, 95% CI 0.83-2.36) was revealed, favoring VR interventions to decrease pain (*z*=4.09; *P*<.001; Figure 5A). For functioning (n=60), an effect size of 1.40 (SE 0.65, 95% CI 0.13-2.67) was calculated (*z*=2.17; *P*=.03; Figure 5B). For mobility (n=24; Figure 5C) and functional capacity (n=21; Figure 5D), effect sizes of 0.49 (SE 0.61, 95% CI −0.71 to 1.68; *z*=0.80, *P*=.42) and 0.34 (SE 0.95, 95% CI −1.52 to 2.20; *z*=0.36; *P*=.72) were revealed, respectively.

## Discussion

### Principal Findings

The main aim of this systematic review was to systematically identify all outcome parameters that are reported in relation to VR in the context of chronic pain management. This review revealed a broad range of outcome variables that are influenced by an intervention of VR technology, with statistically significant results on pain relief and improvements in functioning. These findings indicate that VR not only has applications in acute pain management but also in chronic pain. Over the last decade, medicine has shifted toward the use of innovative tools such as VR. The use of VR technology has expanded from the entertainment industry to clinical medicine and has proven to be cost-effective and efficient [14]. The effects of VR technology have been explored in different settings in medicine, ranging from surgical education to pain management [14]. The use of VR technology in surgical training has become an essential prerequisite for junior physicians before they can actively participate in real surgical operations [85]. Compared with VR technology, trainees who have received conventional surgical training have been found to perform surgery substantially slower and are more likely to cause injury, damage tissue, or fail to progress with surgery [86].

### Comparison With Existing Literature

The management of pain in acute care settings often relies on pharmacological treatments to decrease pathophysiological responses [87]. However, the emergence of VR technology has brought another path for the management of acute pain and has been investigated for the management of burn-induced injuries [14]. As a result, VR technology provides analgesia with minimal side effects [88] and minimal impact on the physical hospital environment [88]. Another approach to the use of VR technology is to augment hypnosis, where patients report lower levels of pain and anxiety [89,90]. In addition to the use of morphine for pain reduction, VR for relaxation has shown to be very effective [91]. In addition, in chronic pain management, the applications of VR technology are very broad, including distraction, cognitive therapy, exercise, and enabling virtual limb movements [32].

Within the field of VR, one of the major classifications is based on the applied materials to induce a virtual experience. Both studies with 3D settings with a computer-generated simulation of 3D environment that makes use of a head-mounted display to entirely replace the real world and computer-generated simulations of 2D environment viewed on a computer or a wall-mounted screen were included [32]. A systematic review concluded that immersive VR technology is more likely to generate pain than nonimmersive (2D) VR technology [30]. As the price of immersive VR equipment continues to decrease, this approach is becoming very affordable and could potentially become a self-management tool for pain relief used by patients in ambulatory and home settings [14]. In this meta-analysis, the type of VR (immersive vs nonimmersive) was not a statistically significant confounder, indicating that VR in general has a beneficial effect on the reported outcome measurements. Further studies are required to confirm these results in chronic pain settings.

Patients affected by chronic pain aim to increase participation in daily living, ranging from work duties and family commitments to social activities and leisure time [92]. Therefore, a complete view of the patient’s functioning and health-related quality of life is needed rather than exclusively assessing the degree of pain relief [93]. A possible framework for organizing and documenting information on functioning and disability is the International Classification of Functioning, Disability, and Health (ICF), which is a classification of health and health-related outcomes [94]. The model conceptualizes functioning as a dynamic interaction between a person’s health condition, personal factors, and environmental factors, as the functioning and disability of an individual occur in this context [95]. Therefore, the ICF provides a multi-perspective, biopsychosocial approach, which is reflected in the multidimensional model [95]. Multiple factors of the ICF model have been found in the reported outcome measurements of this systematic review, whereby mainly bodily functions and structures (eg, pain intensity and sleep), functional disability (eg, walking and balance control), and psychological distress (eg, depression, anxiety, and catastrophizing) were represented. The meta-analyses revealed a statistically significant positive effect of VR on pain-related (95% CI 0.83-2.36; *z*=4.09; *P*<.001) and functioning-related (95% CI 0.13-2.67; *z*=2.17; *P*=.03) outcome measurements. These results are in line with the decrease in pain intensity and disability by VR compared with proprioceptive training and lumbar stabilization exercises in patients with chronic neck pain and chronic low back pain, respectively, as observed in a recent meta-analysis [96]. No statistically significant results were found for functional capacity and mobility, whereby a number of effect sizes were indicative of no effect of VR on these outcome measurements. However, this finding does not exclude the potential added value of VR with another rehabilitation strategy to work on these concepts. Recently, it was suggested that manual therapy in combination with VR could define an entire mind-body intervention that relies on psychological, interoceptive, and exteroceptive stimulations for rebalancing sensorimotor integration and distorted perceptions, including visual and body images [97], denoting the complementary action of several conservative treatments.

### Strengths and Limitations

The main strength of this literature review was the addition of a 3-level meta-analysis to consider the dependency between effect sizes. In the case of dependency, effect sizes are correlated, which inevitably leads to inflation of information and overconfidence in the results of a *standard* meta-analysis [42]. To avoid this inflation, several approaches have been used, among which the selection of only one effect size per study, relying on an average effect size for dependent effect sizes, or dependency could simply be ignored, and the analysis was performed as if the effect sizes were independent [98]. As the aim of this review was to evaluate the effect of VR on all outcome measurements in relation to chronic pain, the authors did not find the stated methods well suited, wherefore a multilevel approach was found to be the most appropriate. Furthermore, this systematic review has certain strengths that secure a minimal risk of potential bias: double-blind screening of the literature, including consensus meetings when needed, and consultation with several databases with the aim of fully representing the existing literature.

Certain limitations should be taken into account as well when interpreting the results of this study. None of the included studies qualified for excellent methodological quality. Of the 41 studies, most of the studies were scored as good on the risk of bias assessment (n=19, 46%), 17 (41%) scored as fair, and only 5 (12%) studies scored as poor. In many of the included studies, the authors mainly focused on the primary outcome variables; in this systematic review, all outcome variables were taken into account (primary and secondary outcome measurements); hence, the results were not always described in full detail for secondary outcomes. Finally, much heterogeneity is present in studies with VR, especially in terms of study design, underlying chronic pain etiologies, VR materials, and VR applications.

### Conclusions

This systematic review explored the outcome measurements that are influenced by VR in patients with chronic pain. A broad range of outcome variables was revealed, whereby an intervention using VR technology can induce pain relief and improvements in functioning. These findings indicate that VR not only has applications in acute pain management but also in chronic pain settings, whereby VR technology might be able to become a promising first-line intervention as a complementary therapy for patients with chronic pain.

## Appendix

Multimedia Appendix 1 The full search strategy for PubMed.

Multimedia Appendix 2 Risk of bias table based on the modified version of the Downs and Black Checklist.

Multimedia Appendix 3 Overview of the effects of virtual reality on several outcome measurements for each study.

## Abbreviations

ICF: International Classification of Functioning, Disability, and Health
PICO: Patient or Population, Intervention, Comparison, and Outcome
PRISMA: Preferred Reporting Items for Systematic Reviews and Meta-Analyses
VR: virtual reality
