# Supplementary material for: Virtual Reality Applications in Chronic Pain Management: Systematic Review and Meta-analysis
Source: JMIR Serious Games. 2022 May 10;10(2):e34402. doi: 10.2196/34402 (PMC9131143; doi:10.2196/34402)
Supplement: Multimedia Appendix 1 [file games_v10i2e34402_app1.docx]

**Supplementary Material 1: Full search strategy in Pubmed**

**Patients:** #1: "chronic pain"[MeSH Terms] OR "pain, intractable"[MeSH Terms] OR "chronic pain patients"[Title/Abstract] OR "chronic pain"[Title/Abstract] OR "persistent pain"[Title/Abstract] OR "intractable pain"[Title/Abstract]

**Intervention**: #2: "virtual reality"[MeSH Terms] OR "virtual reality exposure therapy"[MeSH Terms] OR "virtual reality"[Title/Abstract] OR "virtual reality exposure therapy"[Title/Abstract] OR "VR"[Title/Abstract] OR "augmented reality"[Title/Abstract] OR "artificial intelligence"[Title/Abstract] OR (("computer simulation"[MeSH Terms] OR ("Computer"[All Fields] AND "Simulation"[All Fields]) OR "computer simulation"[All Fields] OR "Simulation"[All Fields] OR "simul"[All Fields] OR "simulate"[All Fields] OR "Simulated"[All Fields] OR "simulates"[All Fields] OR "simulating"[All Fields] OR "simulation s"[All Fields] OR "simulational"[All Fields] OR "simulations"[All Fields] OR "simulative"[All Fields] OR "simulator"[All Fields] OR "simulator s"[All Fields] OR "simulators"[All Fields]) AND "3d environment"[Title/Abstract]) OR "Immersion"[Title/Abstract] OR "computer generated simulation"[Title/Abstract] OR "head mounted display"[Title/Abstract] OR "Cyberspace"[Title/Abstract] OR "virtual technology"[Title/Abstract] OR "simulated reality"[Title/Abstract] OR "simulated environment"[Title/Abstract] OR "virtual simulation"[Title/Abstract] OR "simulation technology"[Title/Abstract]

**Outcomes**: #3: "pain management"[MeSH Terms] OR "psychology"[MeSH Terms] OR "therapeutics"[MeSH Terms] OR "analgesia"[MeSH Terms] OR "exercise"[MeSH Terms] OR "exercise"[MeSH Terms] OR "exercise"[MeSH Terms] OR ((("physical examination"[MeSH Terms] OR ("physical"[All Fields] AND "examination"[All Fields]) OR "physical examination"[All Fields] OR "physical"[All Fields] OR "physically"[All Fields] OR "physicals"[All Fields]) AND ("condition s"[All Fields] OR "conditions"[All Fields] OR "disease"[MeSH Terms] OR "disease"[All Fields] OR "condition"[All Fields])) AND "humans"[MeSH Terms]) OR "catastrophization"[MeSH Terms] OR "catastrophization"[MeSH Terms] OR "movement"[MeSH Terms] OR "motor activity"[MeSH Terms] OR (("acclimatization"[MeSH Terms] OR "acclimatization"[All Fields] OR "adaptation"[All Fields] OR "adaptations"[All Fields] OR "adapt"[All Fields] OR "adaptabilities"[All Fields] OR "adaptability"[All Fields] OR "adaptable"[All Fields] OR "adaptational"[All Fields] OR "adaptative"[All Fields] OR "adapte"[All Fields] OR "adapted"[All Fields] OR "adapting"[All Fields] OR "adaption"[All Fields] OR "adaptions"[All Fields] OR "adaptive"[All Fields] OR "adaptively"[All Fields] OR "adaptiveness"[All Fields] OR "adaptivity"[All Fields] OR "adapts"[All Fields]) AND "psychology"[MeSH Terms]) OR "adaptation, psychological"[MeSH Terms] OR "adaptation, psychological"[MeSH Terms] OR "pharmaceutical preparations"[MeSH Terms] OR "memory"[MeSH Terms] OR "emotions"[MeSH Terms] OR "affect"[MeSH Terms] OR "emotional regulation"[MeSH Terms] OR "emotional adjustment"[MeSH Terms] OR "rumination, cognitive"[MeSH Terms] OR "cognition"[MeSH Terms] OR "sleep"[MeSH Terms] OR "sleep"[MeSH Terms] OR "patient comfort"[MeSH Terms] OR "health"[MeSH Terms] OR "motivation"[MeSH Terms] OR "behavior and behavior mechanisms"[MeSH Terms] OR ("depressive disorder"[MeSH Terms] OR "depression"[MeSH Terms]) OR "depressive disorder"[MeSH Terms] OR "anxiety"[MeSH Terms] OR "anxiety"[MeSH Terms] OR "anxiety"[MeSH Terms] OR "anxiety"[MeSH Terms] OR "personal satisfaction"[MeSH Terms] OR "patient satisfaction"[MeSH Terms] OR "fear"[MeSH Terms] OR "panic"[MeSH Terms] OR "quality of life"[MeSH Terms] OR "quality of life"[MeSH Terms] OR "attention"[MeSH Terms] OR "pain perception"[MeSH Terms] OR "pain"[Title/Abstract] OR "pain intensity"[Title/Abstract] OR "pain reduction"[Title/Abstract] OR "pain management"[Title/Abstract] OR "pain perception"[Title/Abstract] OR "catastrophizing"[Title/Abstract] OR "catastrophization"[Title/Abstract] OR "pain catastrophizing"[Title/Abstract] OR "psychological"[Title/Abstract] OR "psychology"[Title/Abstract] OR "adaptation psychology"[Title/Abstract] OR "coping skills"[Title/Abstract] OR "behavior adaptive"[Title/Abstract] OR "pharmaceutical preparations"[Title/Abstract] OR "therapeutics"[Title/Abstract] OR "medication"[Title/Abstract] OR "emotional"[Title/Abstract] OR "emotions"[Title/Abstract] OR "affect"[Title/Abstract] OR "emotional regulation"[Title/Abstract] OR "emotional adjustment"[Title/Abstract] OR "rumination cognitive"[Title/Abstract] OR "cognition"[Title/Abstract] OR "cognitive"[Title/Abstract] OR "attention"[Title/Abstract] OR "participation"[Title/Abstract] OR "pain relief"[Title/Abstract] OR "comfort"[Title/Abstract] OR "ease"[Title/Abstract] OR "well-being"[Title/Abstract] OR "alleviation"[Title/Abstract] OR "analgesia"[Title/Abstract] OR "patient comfort"[Title/Abstract] OR "health"[Title/Abstract] OR "resilience"[Title/Abstract] OR "self-compassion"[Title/Abstract] OR "activity"[Title/Abstract] OR "exercise"[Title/Abstract] OR "physical activity"[Title/Abstract] OR "training exercise"[Title/Abstract] OR (("physical examination"[MeSH Terms] OR ("physical"[All Fields] AND "examination"[All Fields]) OR "physical examination"[All Fields] OR "physical"[All Fields] OR "physically"[All Fields] OR "physicals"[All Fields]) AND "condition human"[Title/Abstract]) OR "movement"[Title/Abstract] OR "motor activity"[Title/Abstract] OR "stress"[Title/Abstract] OR "stress disorders traumatic"[Title/Abstract] OR "psychological stress"[Title/Abstract] OR "satisfaction"[Title/Abstract] OR "fear of movement"[Title/Abstract] OR "kinesiophobia"[Title/Abstract] OR "anxiety"[Title/Abstract] OR "nervousness"[Title/Abstract] OR "hypervigilance"[Title/Abstract] OR "anxiousness"[Title/Abstract] OR "fear"[Title/Abstract] OR "panic"[Title/Abstract] OR "distraction"[Title/Abstract] OR "function*"[Title/Abstract] OR "acceptance"[Title/Abstract] OR "attention to pain"[Title/Abstract] OR "depressive symptoms"[Title/Abstract] OR "depressive thoughts"[Title/Abstract] OR "depression"[Title/Abstract] OR "depressive disorder"[Title/Abstract] OR "memory"[Title/Abstract] OR "sleep"[Title/Abstract] OR "sleeping habits"[Title/Abstract] OR "motivation"[Title/Abstract] OR ("behavior"[Title/Abstract] AND "behavior mechanisms"[Title/Abstract]) OR "personal satisfaction"[Title/Abstract] OR "patient satisfaction"[Title/Abstract] OR "quality of life"[Title/Abstract] OR "health related quality of life"[Title/Abstract]

**#1 AND #2 AND #3**
