# Supplementary material for: Virtual Reality Applications in Chronic Pain Management: Systematic Review and Meta-analysis
Source: JMIR Serious Games. 2022 May 10;10(2):e34402. doi: 10.2196/34402 (PMC9131143; doi:10.2196/34402)
Supplement: Multimedia Appendix 3 [file games_v10i2e34402_app3.docx]

**Supplementary Material 3: Overview of the effects of VR on several outcome measurements for each study separately.** Abbreviations. NRS: Numeric Rating Scale. VAS: Visual Analogue Scale.

| Outcome Measurements | | | |
| --- | --- | --- | --- |
| Outcome category | **Measurement instrument** | **Significant effect of VR** | **Non-significant effect of VR** |
| Pain | Pain intensity with Numeric Rating Scale | Alemanno et al. (2019) [62]  Fowler et al. (2019) [45]  Gromala et al. (2015) [47]  Jones et al. (2016) [50]: during and after  Matheve et al. (2020) [64]  Monteiro et al. (2015) [65]  Ortiz-Catalan et al. (2016) [77]  Shahrbanian et al. (2008) [52]  Solcà et al. (2020) [82]: VR with SCS  Wiederhold et al. (2014) [53] | Brown et al. (2020) [63]  Garrett et al. (2017) [46]  Harvie et al. (2020) [68]  Herrero et al. (2014) [58] |
|  | McGill Pain Questionnaire | Alemanno et al. (2019) [62]  Guarino et al. (2017) [48]  Mouraux et al. (2017) [76]  Pamment et al. (2016) [51]: back and front synchronous stroking + asynchronous stroking front  Thomas et al. (2016) [66] | Pamment et al. (2016) [51]: asynchronous stroking back |
|  | Short Form McGill Pain Questionnaire | Chau et al. (2019) [73]  Ortiz-Catalan et al. (2016) [77]  Tong et al. (2020) [79] |  |
|  | Visual Analogue Scale | Chau et al. (2019) [73]  Guarino et al. (2017) [48]  Jin et al. (2016) [49]: during the VR intervention  Mouraux et al. (2017) [76]  Phoon Nguyen et al. (2020) [84]: intensity of burning pain  Rezaei et al. (2018) [69]  Sarig-Bahat et al. (2015) [70]  Sarig-Bahat et al. (2018) [71]  Sato et al. (2010) [75]  Solcà et al. (2020) [82]  Tejera et al. (2020) [72]  Thomas et al. (2016) [66]  Tong et al. (2020) [79]  Trujillo et al. (2020) [67]  Wiederhold et al. (2014) [53] | Jin et al. (2016) [49]: after the intervention and during control  Mortensen et al. (2015) [59]  Phoon Nguyen et al. (2020) [84]: overall pain intensity  Sarig-Bahat et al. (2015) [70]: 3 months follow-up |
|  | Wong Baker Faces Scale | Chau et al. (2019) [73] | Phoon Nguyen et al. (2020) [84] |
|  | Brief Pain Inventory (BPI) | Alemanno et al. (2019) [62] except least pain  Guarino et al. (2017) [48]: severity and interference | Garcia-Palacios et al. (2014) [57]  Garrett et al. (2017) [46] |
|  | Douleur Neuropathique 4 Questions (DN4 Questionnaire) | Mouraux et al. (2017) [76] |  |
|  | Defense and Veterans Pain Rating Scale | Darnall et al. (2020) [81] |  |
|  | Pain intensity | Amin et al. (2017) [44]  Rutledge et al. (2019) [78]  Thomas et al. (2016) [66]  Wiederhold et al. (2014) [53]  Wiederhold et al. (2014) [54] | Amin et al. (2017) [44] |
|  | Weighted pain distribution | Ortiz-Catalan et al. (2016) [77] |  |
|  | Pain Rating Index | Ortiz-Catalan et al. (2016) [77] |  |
|  | Self-Administered Leeds Assessment of Neuropathic Symptoms and Signs (S-LANSS) |  | Garrett et al. (2017) [46] |
|  | Child Daily Questionnaire | Griffin et al. (2020) [80]: pain, fear, avoidance, functional limitations | Griffin et al. (2020) [80]: activity engagement, pain reactivity |
| Psychological | Pain Outcomes Questionnaire-VA Fear |  | Fowler et al. (2019) [45] |
|  | Pain Outcomes Questionnaire-VA Negative affect |  | Fowler et al. (2019) [45] |
|  | Pain Catastrophizing Scale | Darnall et al. (2020) [81]  Fowler et al. (2019) [45]  Matheve et al. (2020) [64]  Tejera et al. (2020) [72]  Trujillo et al. (2020) [67] |  |
|  | Chronic Pain Coping Inventory | Garcia-Palacios et al. (2014) [57]: persistence | Garcia-Palacios et al. (2014) [57]: guarding, resting, asking for assistance, relaxation, exercise, coping self-statements, seeking social support |
|  | Pain Self-efficacy | Darnall et al. (2020) [81] |  |
|  | Pain anxiety symptoms scale | Tejera et al. (2020) [72] |  |
|  | Activities- Self-efficacy | Herrero et al. (2014) [58] |  |
|  | Activities-Motivation | Herrero et al. (2014) [58] |  |
|  | Beck Depression Inventory | Alemanno et al. (2019) [62] | Guarino et al. (2017) [48] |
|  | Beck Depression Inventory II | Botella et al. (2013) [55]  House et al. (2016) [83] | Garcia-Palacios et al. (2014) [57] |
|  | Positive and Negative Affect Schedule | Botella et al. (2013) [55] |  |
|  | Mood state with pictorial scale | Herrero et al. (2014) [58] |  |
|  | Profile of Mood States |  | Monteiro et al. (2015) [65] |
|  | Anxiety thermometer |  | Brown et al. (2020) [63] |
|  | Fear of falling using VAS | Collado-Mateo et al. (2017) [56] |  |
|  | Fear of Daily Activities Questionnaire |  | Fowler et al. (2019) [45] |
|  | Subjective Units of Distress Scale |  | Guarino et al. (2017) [48] |
|  | State-Trait Anxiety Inventory |  | Guarino et al. (2017) [48] |
|  | Tampa Scale for Kinesiophobia | Tejera et al. (2020) [72]: 3 months follow-up | Matheve et al. (2020) [64]  Sarig-Bahat et al. (2015) [70]  Sarig-Bahat et al. (2018) [71]  Tejera et al. (2020) [72]: post intervention and 1 month follow-up |
|  | Fear-avoidance beliefs questionnaire | Tejera et al. (2020) [72]: 3 months follow-up | Tejera et al. (2020) [72]: post intervention and 1 month follow-up |
|  | Fatigue (NRS) |  | Herrero et al. (2014) [58] |
|  | Brief Fatigue Inventory |  | Mortensen et al. (2015) [59] |
|  | Engagement | Shahrbanian et al. (2008) [52] |  |
|  | Anxiety (NRS) | Herrero et al. (2014) [58] |  |
|  | Anger (NRS) |  | Herrero et al. (2014) [58] |
|  | Joy (NRS) | Herrero et al. (2014) [58] |  |
|  | Sadness (NRS) | Herrero et al. (2014) [58] |  |
|  | Surprise (NRS) | Herrero et al. (2014) [58] |  |
|  | Calmness (NRS) | Herrero et al. (2014) [58] |  |
|  | Vigor/Energy (NRS) | Herrero et al. (2014) [58] |  |
|  | Illusory agency (NRS) | Matamala-Gomez et al. (2016) [74] |  |
| Functioning | Pain Outcomes Questionnaire-VA Interference daily living |  | Fowler et al. (2019) [45] |
|  | Timed up and go test | Collado-Mateo et al. (2017) [56] |  |
|  | Functional reach | Collado-Mateo et al. (2017) [56] |  |
|  | Patient-specific Functional Scale | Fowler et al. (2019) [45] |  |
|  | Fulg-Meyer Assessment |  | House et al. (2016) [83] |
|  | Chair-stand test | Villafaina et al. (2020) [60] |  |
|  | Six-minute walk test | Villafaina et al. (2020) [60] |  |
|  | 10 step stair test |  | Villafaina et al. (2020) [60] |
|  | Roland and Morris Disability Questionnaire (RMDQ) | Alemanno et al. (2019) [62] | Matheve et al. (2020) [64] |
|  | Activities of Daily Living | Ortiz-Catalan et al. (2016) [77] | Alemanno et al. (2019) [62]  Mortensen et al. (2015) [59] |
|  | Fibromyalgia Impact Questionnaire | Botella et al. (2013) [55]  Garcia-Palacios et al. (2014) [57] |  |
|  | Neck Disability Index | Rezaei et al. (2018) [69]  Sarig-Bahat et al. (2015) [70]  Sarig-Bahat et al. (2018) [71]  Tejera et al. (2020) [72] |  |
|  | Sleep (NRS) | Ortiz-Catalan et al. (2016) [77] |  |
|  | Chedokee Arm and Hand Activity Inventory-9 |  | House et al. (2016) [83] |
|  | Upper Extremity Functional Index 20 |  | House et al. (2016) [83] |
|  | Jebsen Hand Function Test- affected side |  | House et al. (2016) [83] |
|  | Functional capacity Sit & Stand |  | Monteiro et al. (2015) [65] |
|  | Functional capacity Sit |  | Monteiro et al. (2015) [65] |
| Functional capacity | Y-balance test | Rezaei et al. (2018) [69] |  |
|  | Step Test | Sarig-Bahat et al. (2015) [70] |  |
|  | Repetition Index | Alemanno et al. (2019) [62] |  |
|  | Balance (Wii Balance Board) |  | Monteiro et al. (2015) [65] |
|  | Composite value | Rezaei et al. (2018) [69] |  |
|  | Clinical Test of Sensory Integration of Balance | Collado-Mateo et al. (2017) [56]: eyes closed on an unstable surface | Collado-Mateo et al. (2017) [56]: other balance scales) |
| Mobility | Range of Motion | Tejera et al. (2020) [72]: rotation | Sarig-Bahat et al. (2018) [71] |
|  | Range of Motion – Lateroflexion |  | Tejera et al. (2020) [72] |
|  | Range of Motion – Flexion/Extension |  | Tejera et al. (2020) [72] |
|  | Pain Outcomes Questionnaire-VA Interference mobility |  | Fowler et al. (2019) [45] |
|  | Rotation | Alemanno et al. (2019) [62] |  |
| Neuropsychological | Trail Making Test |  | House et al. (2016) [83] |
|  | Neuropsychological Assessment Battery |  | House et al. (2016) [83] |
|  | Hopkins Verbal Learning Test-Revised |  | House et al. (2016) [83] |
|  | Brief Visuospatial Memory Test Revised | House et al. (2016) [83] | House et al. (2016) [83] |
|  | Resting brain dynamics (EEG signals) | Villafaina et al. (2019) [61]: power in the beta-3 frequency band, frontal, parietal, temporal and occipital areas |  |
| Quality of Life | Quality of Life Index | Garcia-Palacios et al. (2014) [57] |  |
|  | Self-rated health status in the European Life Quality Questionnaire | Sarig-Bahat et al. (2018) [71] |  |
|  | Short Form Health Survey | Alemanno et al. (2019) [62] |  |
| Other sensations | Phantom sensations | Rutledge et al. (2019) [78] |  |
|  | Pressure Pain Treshold | Tejera et al. (2020) [72]: left (1 month follow-up) | Tejera et al. (2020) [72]: right |
|  | Movement-evoked pain treshold |  | Harvie et al. (2020) [68] |
|  | Cold pain treshold |  | Shahrbanian et al. (2008) [52] |
|  | Hot pain treshold | Shahrbanian et al. (2008) [52] |  |
|  | Skin temperature | Wiederhold et al. (2014) [54] |  |
